# Impact of traumatic events incurred by asylum-seekers on mental health and utilization of medical services

**DOI:** 10.1186/s13049-019-0665-8

**Published:** 2019-09-06

**Authors:** Maya Siman-Tov, Moran Bodas, Alex Wang, Michael Alkan, Bruria Adini

**Affiliations:** 1 Emergency management & disaster medicine department, School of Public Health, Sackler Faculty of Medicine, Tel Aviv University, Tel Aviv, Israel; 2 National Center for Trauma & Emergency Medicine Research, the Gertner Institute for Health Policy and Epidemiology, Tel Hashomer, Ramat Gan, Israel; 3 Medical School for International Health, Ben-Gurion University of the Negev, Beer Sheva, Israel

**Keywords:** Asylum-seekers, Traumatic events, Torture, Utilization of medical services, Post-traumatic stress disorder

## Abstract

**Background:**

Asylum-seekers from Africa immigrate to Israel through the Sinai desert and are often exposed to traumatic events.

**Objective:**

To identify the scope and types of medical services required by asylum-seekers and the relationship between delayed medical care to development of post-traumatic stress disorder (PTSD) and overutilization of medical services.

**Methods:**

Asylum-seekers that entered Israel between 2009 and 2012 who utilized the Open Clinic of Physicians for Human Rights were interviewed to record their experiences in the Sinai, and document the traumatic events they were exposed to, their medical diagnoses, and clinic visits. Linkages between diagnoses to exposure to traumatic events and period of time until presentation to the clinic were investigated.

**Results:**

Male vs female asylum-seekers visited the clinic more times (24% vs 15% respectively, utilized > 5 visits). Higher ransom and longer periods in Sinai correlated with higher number of clinic visits and PTSD. Asylum-seekers with PTSD versus other medical complaints approached the clinic more times (> 5 visits). Asylum-seekers that approached the clinic closer to their arrival time (up to 18 months from arrival) versus a later period (> 18 months) presented a significantly lower prevalence of PTSD (3.4 and 40.5% respectively; *p* < 0.001) and lower utilization of clinic’s services (p < 0.001).

**Conclusions:**

PTSD among asylum-seekers appears to be associated more with length of exposure to stressful events than number/types of traumatic events and with delay in receiving medical care. Improving access to medical care may reduce asylum-seekers’ development of PTSD and lead to lower utilization of services.

## Background

Displaced populations including asylum-seekers and refugees were recognized as a global challenge since WWII but have been highlighted as a world-wide concern in the past decade [1, 2]. Today there are over 70.8 million forcibly displaced people worldwide that are recognized by the United Nations High Commissioner for Refugees [3].

Over 60,000 asylum-seekers entered Israel between the years 2006 to 2012, primarily from African countries such as Sudan and Eritrea [4, 5]. The number of asylum-seekers significantly dropped after 2012 as a barrier fence between Israel and the Sinai desert was constructed, substantially limiting their ability to cross the border [5, 6]. As of 2017, less than 36,000 asylum seekers remained in Israel [4]. These individuals are often regarded by authorities as undocumented migrant workers or “infiltrators” that are not entitled to the benefits, including medical services, that are legally provided to refugees [4, 6].

Asylum seekers that entered Israel through the Sinai desert were often exposed to extremely stressful events, including violence, torture, and captivity [5, 7]. Many of them were exposed to gender-based violence and human trafficking [8, 9]. Traumatic events among migrants and asylum-seekers have been identified as triggers for the development of both physical and mental health problems, specifically Post-Traumatic Stress Disorder (PTSD), depression and anxiety [10–13].

Marginalized populations including asylum-seekers have relatively limited access to essential medical services and difficulty integrating into healthcare systems. These populations often suffer from psychiatric conditions, which have widely been associated with increased utilization of medical services, and may result in the host countries’ perception that their ability to provide medical services to the asylum-seekers exceeds their capacity [1, 10, 14]. These barriers of asylum-seekers to care may stem from fear of deportation, detainment in camps lacking in basic healthcare facilities, culture-specific barriers, ineligibility to obtain care in public services, or lack of funding to be treated in the private sector [1, 15].

The volunteer-based “Open Clinic” in Tel Aviv run by Physicians for Human Rights Israel (PHR) [16–18] has provided care to the marginalized and uninsured for over 20 years and has been one of the primary clinics providing health-services for the asylum seekers in Israel [17, 19]. The clinic is set within the metropolitan area and serves the asylum-seekers that reside in the urban setting. In 2012, 1150 asylum seekers were interviewed about their experiences regarding their stay in the Sinai desert. These traumatic experiences in combination with the difficulties of cultural acclimation contributed largely to depression and mental illness [7, 18]. The open clinic employs senior physicians that though are not specialists in psychiatry, are highly experienced and have been trained in diagnosing mental health symptomatology. Upon such diagnoses, the patients, including those suffering from Post Trauma Stress Disorder (PTSD) were immediately referred to treatment in a designated clinic ran by the Ministry of Health. This was deemed as important, especially considering the uncertainty of the asylum-seekers’ situation and their fear of the future.

As the government of Israel has not made any final decisions regarding the patriation of the asylum seekers, their legal and thus medical status remain unresolved for the last decade [19]. This may be reflected in an increase of patients seen at the clinic since 2015 who had not before used the services of the clinic. The clinical observation of a high frequency of PTSD symptoms, long after the traumatic events, hinted that this group may be different, and more information is needed to elucidate this difference.

### Importance

Provision of universal health coverage is a core element of the Sustainable Development Goals [2]. Ensuring the capacity of host countries to provide essential medical care to asylum-seekers is dependent on tailoring the services to their specific medical needs [1, 15]. Identifying factors that aggravate medical needs and designing measures that may decrease the development of health problems, will contribute to cost-benefit, effective healthcare provision. Therefore, there is a need to understand the impact of traumatic events on the utilization of medical services [12, 14].

### Goals of this investigation

To identify the scope and types of medical services utilized by asylum-seekers and the relationship between delayed medical care to the development of PTSD.

## Methods

A retrospective cohort study of 861 asylum-seekers who entered Israel between 2009 and 2012 and received medical care at the Open Clinic of Physicians for Human Rights was performed. The asylum-seekers that approach the clinic are those that do not have medical insurance or eligibility to medical coverage, and thus they seek medical services at the clinic. Following the request of the UNHCR (the United Nations High Commissioner of Refugees) to document traumatic events that asylum-seekers were exposed to during their flee to places of refuge, all patients that presented to the clinic up to 2012 were screened by the clinic’s staff via a brief questionnaire about their experiences in the Sinai desert. They were asked about length of stay in Sinai, deprivation of vital elements (lack of food, water, shelter, medical care etc.), exposure to torture of themselves or of others (beating, kidnapping, ransom etc.), and sum of ransom (if paid). Those who screened positive (i.e. were exposed to any type of torture), were interviewed at length in their native languages of Tigrinya or Arabic. Patients who were not exposed to any traumatic event during their stay in the Sinai desert were excluded from the study. The asylum-seekers were further diagnosed and treated by the clinic’s physicians. Asylum-seekers that presented from 2012 to 2016 were similarly screened through interviews by the physicians at the clinic, using language interpreters. Data from the interviews and from chart reviews were collected and anonymized.

The research protocol was approved by the ethics committees of the Ben-Gurion University of the Negev number 20164 from February 2, 2016. Following this approval, the retrospective study was initiated, based on the files of the asylum-seekers that are kept in the clinic.

Data collected included demographic characteristics, types of traumatic experiences in the Sinai desert, and clinical information including date of presentation at the clinic, diagnoses, number of visits to the clinic, and medical services that were utilized, as follows:

### Demographic characteristics

Gender, age (<20 years old, 21–30 years old, 31–40 years old and 41+ years old), country of origin and date of arrival to Israel.

### Experiences in Sinai desert

Number of days spent in the Sinai desert (< 7 days, 7–20 days, 21–60 days, > 60 days), ransom paid in order to be released after kidnap (< 2500 USD [limited], 2500–4000 USD [intermediate] and > 4000 USD [high]), type of traumatic event (gunshot wounding, physical assault, sexual assault, imprisonment, or other mistreatment).

The modified Harvard Trauma Questionnaire (MHTQ) has been extensively used in the past 25 years to characterize exposures to traumatic events, particularly in migrant asylum-seeker and refugee populations, which include abduction, imprisonment, physical and sexual violence among others [10, 11]. Consequently, these events have been identified as triggers for the development of both physical and mental health problems, specifically PTSD and functional distress in this population [12, 13]. The MHTQ is also used to diagnose symptoms of PTSD and functional distress in these populations [12, 13].

Based on this data, the modified Harvard Trauma Score (MHTS) was calculated to the asylum seekers and categorized into three categories from ‘Low’ (0–1 type of torture), ‘Moderate’ (2 types) and ‘High’ (3–5 types).

### Clinical data

Per chart review, medical diagnoses were categorized into the following categories: pregnancy, sexual transmitted disease and gynecology, skin, gastrointestinal, physical trauma, PTSD, orthopedic non-trauma, respiratory, ocular and internal diseases including liver, diabetes, hypertension, and tuberculosis. The category of orthopedic non-trauma includes chronic conditions that did not involve a recent injury, such as pain stemming from old injuries. Utilization of the clinic was separately categorized based on number of visits (1 visit, 2–4 visits, and 5+ visits). A diagnosis of PTSD was made in patients who presented with three or more of the criteria of the Diagnostic and Statistical Manual of Mental Disorder - 5 (DSM-5) [12, 20]. The DSM-5 recognizes that PTSD is derived from experience or threat of traumatic events, including torture, injuries, sexual assaults or other violent behavior. The actual exposure of the individual to such events and/or witnessing them and/or exposure of loved ones to such atrocities, may cause clinical stress and incapacity of the individual to function properly [20]. The open clinic’s physicians are the ones that diagnosed PTSD among the asylum-seekers, dependent on DSM-5 [12, 20].

### Data analysis

Descriptive statistics were used to analyze the characteristics of the sample. Linkages between diagnoses to exposure to stressful events and period of time until presentation to the clinic, number of visits to the clinic and different diagnoses were investigated using chi square test. All statistical analyses were performed using SPSS software version 25. *P*-values lower than 0.05 were considered to be statistically significant.

## Results

Table 1 presents the demographic characteristics, traumatic experiences in the Sinai desert, clinical data and the calculated MHTQ. The majority of patients were male (63%), under 30 (67%), and from Eritrea (84%). About 64% stayed in the Sinai desert up to 20 days. Almost 64% paid significant amounts for ransom.

**Table 1:** Demographic, experiences in Sinai desert and clinic characteristics of the asylum-seekers

	*N* = 861
Male *N (%)*	542 (63.2%)
*Unknown- 3*	
Age *(years) mean ± SD*	29.03 ± 7.71
<20	60 (7.0%)
21–30	517 (60.1%)
31–40	213 (24.8%)
41+	70 (8.1%)
*Unknown- 1*	
(Origin) country of origin *N (%)*	
Eritrea	724 (84.3%)
Sudan	102 (11.9%)
Other	33 (3.8%)
*Unknown- 2*	
Number of days spent in the Sinai desert	11 [5–30], (0–520)^a^
<6	234 (30.0%)
7–20	264 (33.8%)
21–60	178 (22.8%)
61+	105 (13.4%)
*Unknown- 80*	
Amounts of ransom (US$)	3000 [2500-3400], (0–43,500)^a^
Limited amount (<2500)	265 (36.5%)
Intermediate amount (2500-4000)	352 (48.5%)
High amount (> 4000)	109 (15.0%)
*Unknown- 134*	
Type of torture	
Gunshot wounds *Unknown- 285*	224 (38.9%)
Physical assault *Unknown- 123*	307 (41.6%)
Sexual assault *Unknown- 265*	39 (6.5%)
Other mistreatment *Unknown- 257*	64 (10.6)
Imprisonment *Unknown- 194*	604 (90.6%)
Modified Harvard Trauma Score	
Low	499 (58.0%)
Moderate	279 (32.4%)
High	83 (9.6%)
Time till arrival to clinic	
<6 months	206 (27.4%)
6–18 months	267 (35.5%)
>18 months	279 (37.1%)
Diagnosis	
Pregnancy	203 (23.6%)
STD + Gynecology	61 (7.1%)
Skin	53 (6.2%)
Gastrointestinal	93 (10.8%)
Trauma	93 (10.8%)
Post Traumatic Syndrome Disorder	134 (15.6%)
Orthopedic- non-trauma	83 (9.6%)
Respiratory	86 (10.0%)
Eye	33 (3.8%)
Liver, diabetes, Blood pressure, Tuberculosis	22 (2.6%)
Number of clinic visits	
1	295 (42.3%)
2–4	260 (37.2%)
5+	143 (20.5%)
*Unknown- 163*	

^a^Data is presented as mean ± standard deviation, N(%), median [p25-p75] and (Minimum-Maximum)

### Visits to the clinic

**Fig. 1:**
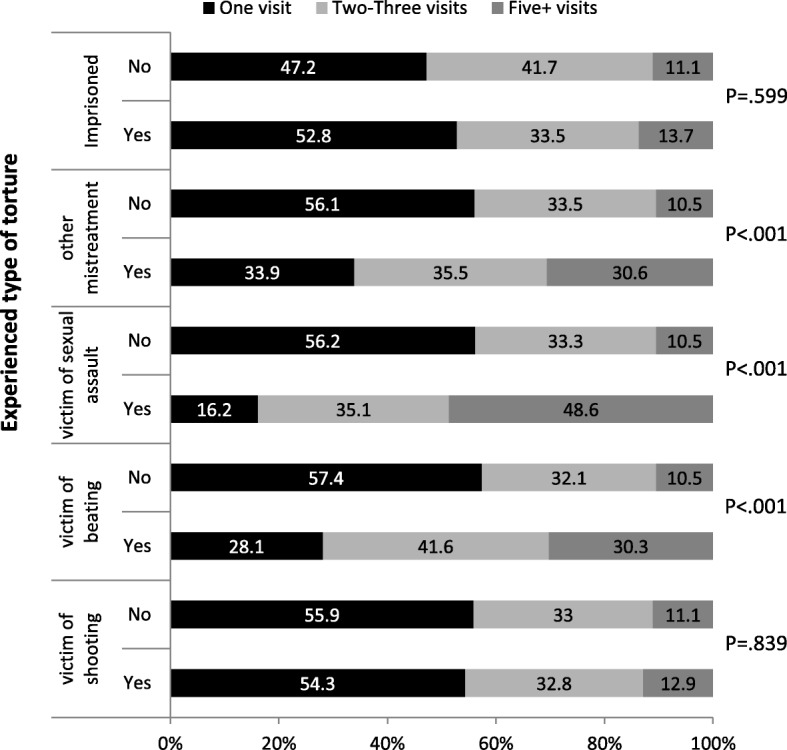
Number of clinic visits: Distribution (in percentage) by type of torture

Utilization of the clinic was plotted against demographic characteristics, experiences in the Sinai desert and diagnoses, presented in Fig. 1 and Table 2. Males presented more times (> 5 visits) than females (24% vs 15%, respectively, *p* = .037). Those who paid the highest ransom and/or spent more than 2 months in the Sinai desert presented more frequently to the clinic. Patients with non-traumatic orthopedic complaints (43%) and PTSD (35%) presented at the clinic more times (> 5 visits) than those with other medical complaints (Table 2).

**Table 2:** Number of clinic visits by demographic, experiences in Sinai desert and diagnosis

	Number of clinic visits				
	1	2–4	5+	χ^2^	*P*-value
Gender					
Male	41.1%	35.4%	23.5%	6.60	.037
Female	44.5%	40.2%	15.4%		
Age (Years)					
<20	52.3%	40.9%	6.8%	25.71	<.001
21–30	44.8%	38.9%	16.4%		
31–40	37.9%	32.2%	29.9%		
41+	29.1%	38.2%	32.7%		
Country of origin					
Eritrea	42.5%	38.1%	19.4%	6.41	.171
Sudan	44.4%	29.6%	25.9%		
Other	22.2%	44.4%	33.3%		
Days spent in the Sinai desert					
<6	54.4%	34.5%	11.1%	39.50	<.001
7–20	51.8%	36.7%	11.6%		
21–60	40.4%	37.7%	21.9%		
61+	23.8%	44.6%	31.7%		
Amounts of ransom ($US)					
Limited amount (< 2500)	52.1%	33.5%	14.4%	14.80	.005
Intermediate amount (2500-4000)	49.8%	38.2%	12.0%		
High amount (> 4000)	36.1%	37.1%	26.8%		
Modified Harvard Trauma Score					
Low	41.5%	38.4%	20.1%	1.55	.818
Moderate	41.9%	36.0%	22.0%		
High	47.8%	34.8%	17.4%		
Time till arrival to clinic					
<6 months	52.8%	32.0%	15.2%	65.96	<.001
6–18 months	53.7%	35.8%	10.6%		
>18 months	24.8%	41.75	33.5%		
Diagnosis					
Pregnancy	50.6%	45.5%	3.9%	117.35	<.001
STD / Gynecology	49.0%	29.4%	21.6%		
Skin	61.0%	22.0%	17.1%		
Gastrointestinal	51.5%	35.3%	13.2%		
Trauma	48.1%	23.4%	28.6%		
Post Traumatic Syndrome Disorder	16.9%	48.5%	34.6%		
Orthopedic- non-trauma	24.7%	32.5%	42.8%		
Respiratory	49.2%	42.4%	8.5%		
Eye	62.5%	29.2%	8.3%		
Liver, diabetes, Blood pressure, Tuberculosis	58.8%	23.5%	17.6%		

Logistic regression model was used to assess the factors associated with a higher number of clinic visits (> 5) compared to less than 5 visits. The variables entered to the model were demographics and experiences in the Sinai desert. The models suggested that only older asylum-seekers and longer stays in the Sinai desert (> 20 days) predict a higher utilization of health services, as reflected in the number of clinic visits (Table 3).

**Table 3:** Logistic regressions identifying variables associated with 5 or more clinic visits by demographic and experiences in Sinai desert

	OR 95% CI	*P*-value
Gender		
Male	1.12 (0.61–2.05)	.714
Female	1	
Age (Years)		
<20	1	
21–30	5.56 (0.72–42.80)	.100
31–40	10.25 (1.27–82.49)	.029
41+	13.09 (1.41–121.41)	.024
Country of origin		
Eritrea	0.37 (0.08–1.66)	.196
Sudan	0.61 (0.11–3.34)	.567
Other	1	
Days spent in the Sinai desert		
<6	1	
7–20	1.31 (0.62–2.76)	.478
21–60	2.36 (1.05–5.31)	.039
61+	4.11 (1.45–11.68)	.008
Amounts of ransom ($US)		
Limited amount (<2500)	1	
Intermediate amount (2500-4000)	1.47 (0.60–3.59)	.400
High amount (>4000)	0.85 (0.44–1.64)	.619
Modified Harvard Trauma Score		
Low	1	
Moderate	0.67 (0.37–1.21)	.181
High	0.63 (0.25–1.54)	.307
Time till arrival to clinic		
<6 months	1	
6–18 months	0.79 (0.42–1.50)	.467
>18 months	1.05 (0.50–2.21)	.897

Asylum-seekers that were exposed to physical violence, sexual assault or other mistreatment, presented to the clinic more times (> 5 visits) compared with those who were not afflicted with such traumatic events (Fig. 1).

Asylum-seekers that sought care at a later date in relation to their arrival time to Israel (> 18 months) presented to the clinic more (> 5 visits) compared to those that sought care within the first 18 months from arrival) (34, and 11%, respectively; *p* < 0.001). (Table 2).

### Post-traumatic stress disorder

The incidence of PTSD according to demographic characteristics, experiences in the Sinai desert and utilization of medical services in the clinic is presented in Table 4. PTSD was found to be more prevalent among men versus women (21% vs. 6% respectively, *p* < 0.001), as well as among older (ages > 31) compared to younger (< 20) patients (40% vs. 18%respectively *p* = .005). A longer period spent in the Sinai desert correlated with the diagnosis of PTSD more frequently (44% for 61+ days vs 2% for less than 7 days, *p* < 0.001). A higher amount of ransom paid while in the Sinai desert correlated with PTSD (31% for high amounts, vs 2% for a limited amount, *p* < 0.001).

**Table 4:** PTSD distribution by demographic, experiences in Sinai desert and clinic characteristics and results of logistic regressions identifying variables associated with PTSD

	PTSD diagnosis				OR (95%CI)
	Yes	No	χ^2^	*P*-value	for PTSD
Gender					
Male	21.2%	78.8%	35.02	<.001	5.42 (2.07–14.20)**
Female	6.0%	94.0%			1
Age (Years)					
<20	3.3%	96.7%	12.93	.005	1
21–30	14.3%	85.7%			0.92 (0.16–5.21)
31–40	21.1%	78.9%			1.09 (0.17–7.95)
41+	18.6%	81.4%			0.21 (0.02–2.07)
Country of origin					
Eritrea	16.3%	83.7%	1.71	.425	1.93 (0.20–18.35)
Sudan	11.8%	88.2%			2.96 (0.21–42.09)
Other	12.1%	87.9%			1
Days spent in the Sinai desert					
<6	1.7%	98.3%	152.03	<.001	1
7–20	3.4%	96.6%			1.23 (0.28–5.45)
21–60	19.7%	80.3%			3.47 (0.81–14.81)
61+	43.8%	56.2%			2.75 (0.51–14.90)
Amounts of ransom (US$)					
Limited amount (<2500)	1.9%	98.1%	85.01	<.001	1
Intermediate amount (2500-4000)	7.1%	92.9%			12.71 (2.71–59.57)**
High amount (> 4000)	31.2%	68.8%			6.00 (1.57–22.86)**
Modified Harvard Trauma Score					
Low	15.6%	84.4%	4.06	.131	1
Moderate	17.6%	82.4%			0.76 (0.32–1.81)
High	8.4%	91.6%			0.78 (0.21–2.90)
Time till arrival to clinic					
<6 months	3.4%	96.6%	179.49	<.001	1
6–18 months	2.2%	97.8%			0.50 (0.13–1.95)
>18 months	40.5%	59.5%			7.79 (2.41–25.23)**
Number of clinic visits					
1	7.5%	92.5%	45.23	<.001	1
2–4	24.2%	75.8%			6.09 (2.23–16.64)***
5+	31.5%	68.5%			3.32 (1.08–10.25)*

*p < .05 **p < .01 ***p < .001

**Fig. 2:**
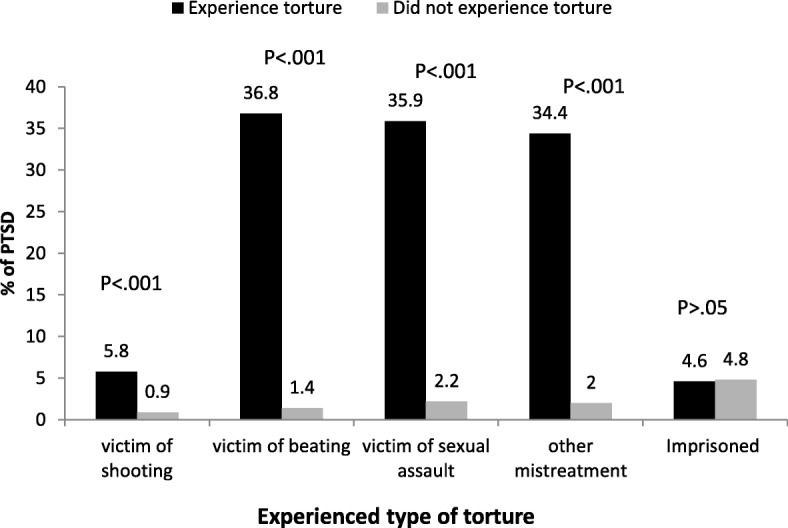
PTSD distribution (in percentage) by type of torture

Although no association was found between PTSD and MHTS directly, examination of separate types of abuse, except for imprisonment, correlated with a diagnosis of PTSD (Fig. 2).

Asylum-seekers that sought care earlier presented with a significantly lower prevalence of PTSD (3.4% within the first 6 months, 2.2% within the first 18 months and 40.5% after 18 months, *p* < 0.001). They also presented lower prevalence of non-trauma orthopedic complaints (4.9, 4.5 and 19.4%, respectively; *p* < 0.001) but a significantly higher prevalence of pregnancies (28.2, 37.1 and 7.9% respectively; *p* < 0.001).

A logistic regression model was used to assess factors associated with PTSD. The variables entered to the model were demographic, experiences in the Sinai desert and clinic visits. The model suggested that gender (male OR 5.42 95% CI 2.07–14.20), a higher amount of ransom (moderate OR 12.71 95% CI 2.71–59.57 and high OR 6.00 95% CI 1.57–22.86), a higher number of clinic visits (2–4 visits OR 6.09 95% CI 2.23–16.64 and ≥ 5 visits OR 3.32 95% CI 1.08–10.25) and delay in presentation to the clinic (OR 7.79 95% CI 2.41–25.23) predict PTSD. (Table 4) (Fig. 2).

## Discussion

Limited access to healthcare services and development of varied mental health symptomatology have been identified as characteristic of displaced populations [21–23]. Individuals with witnessed traumatic events frequently display mental health disorders [24, 25]. Similar to previous studies, the current study suggests that the combination of torture in the context of acculturation may result in increased signs of mental illness [10–12].

Contrary to numerous prior studies that suggest a higher prevalence of PTSD among women [13, 23, 26], the current study identifies a higher prevalence among men. This may be derived from a greater perceived risk of being detained and deported, which under the current sociopolitical climate and passage of the Israeli “Anti-Infiltration” law, has allowed for the construction of several detention facilities that only accommodate male asylum seekers [5]. It may also result from the expectation of men to be primary providers, so that the inability to appropriately function in this role may aggravate their mental health. It should though be noted that not only the traumatic events themselves, but also the harsh life encountered by the asylum-seekers in their origin countries, their current financial loss and diminished hope for the future, may likewise contribute to significant psychologic damage.

It was surprising that PTSD among asylum-seekers appears to be associated more with the overall length of exposure to stressful events rather than the actual number of traumatic episodes. Several studies presented that the severity and number of traumatic events encountered by the asylum seekers are related to the development of PTSD [10, 11, 15]. The current study proposes that exposure to even one type of stressful traumatic event is sufficient to contribute to the onset of PTSD. These findings strengthen the idea that despite the number of traumatic stressors, individuals exposed to protracted periods of displacement may have increased PTSD and mental health problems [5, 23].

Similarly, the need to pay higher ransoms in order to be released from captivity was found to be related with higher levels of PTSD. Demands for ransom are a common practice in areas of conflict around the globe [26, 27]. As displaced populations often do not have strong support systems that they can approach to pay the required ransom, this demand presents a highly stressful traumatic event. Social support has been found to be a protective factor against development of PTSD, even in cases of exposure to traumatic events [5]. The lack of these support systems most probably contributes to an enhanced vulnerability of this population.

Patients with delayed medical care had a higher utilization of healthcare services over time. This suggests that any perceived or actual barrier to care ultimately increases a society’s healthcare burden. Given the current context where numerous countries have developed increasingly restrictive policies concerning access to healthcare for displaced populations, this may have a detrimental impact financially [27–30]. Given the increasing global burden of displaced populations around the world, the need for improved accessibility must be highlighted. Enhancing access to medical services may significantly decrease the development of PTSD among asylum-seekers and ultimately less resources will be required to treat this population [15].

The study has several limitations. The lack of information concerning asylum seekers who did not present to the PHR clinic seeking medical care may be confounding. In addition, this study retrospectively compares groups of patients who are demographically similar, but were assessed by different medical providers.

To conclude, it is vital to understand risk and protective factors of asylum-seekers in relation to access to medical care, to tailor the medical treatment to their needs and ease their acclimatization in the receiving country. Consequently, it may be possible to reduce their vulnerability and development of PTSD and achieve a more efficient utilization of healthcare resources. Implementing outreach measures that integrate vulnerable populations into the healthcare and social framework earlier would be expected to alleviate suffering and achieve a more successful absorption of displaced populations in societies world-wide.

## Data Availability

The data generated or analysed during this study are included in this published article; if any additional data is requested, please approach the corresponding author (adini@tauex.tau.ac.il); nonetheless, restrictions apply concerning these data, as they were used under specific approval of the Open Clinic of Physicians for Human Rights.

## Abbreviations

DSM-5: Diagnostic and Statistical Manual of Mental Disorders 5
MHTQ: modified Harvard Trauma Questionnaire
MHTS: modified Harvard Trauma Score
PHR: Physicians for Human Rights
PTSD: Post Traumatic Stress Disorder
UNHCR: United Nations High Commissioner of Refugees
